# Incidental Detection of a Small, Non-spiculated Breast Cancer During Preoperative Evaluation for an Unruptured Cerebral Aneurysm With Gradual Enlargement Over 16 Years: A Case Report

**DOI:** 10.7759/cureus.107905

**Published:** 2026-04-28

**Authors:** Ayumu Yamaoka, Takeru Nishino, Ryohei Saito, Katsuya Komatsu, Nobuhiro Mikuni

**Affiliations:** 1 Department of Neurosurgery, Sapporo Medical University, School of Medicine, Sapporo, JPN

**Keywords:** cerebrovascular disease, comprehensive assessment, extracranial incidental findings, japanese geriatrics, preoperative evaluation

## Abstract

In older patients with unruptured intracranial aneurysms that have been followed over long periods, careful assessment of operative tolerance and comorbidities becomes an important component of treatment decision-making. Although cross-sectional imaging performed for procedural planning may occasionally reveal extracranial incidental findings, neurosurgical evaluation is typically focused on intracranial pathology.

We report a case of an asymptomatic, small, non-spiculated breast cancer incidentally detected during preoperative evaluation for a middle cerebral artery (MCA) aneurysm that had demonstrated gradual enlargement over 16 years. A woman in her 70s was diagnosed with an unruptured cerebral aneurysm at the bifurcation of the right MCA during routine brain screening. Serial magnetic resonance angiography (MRA) demonstrated a gradual increase in aneurysm height, with the development of blebs, prompting surgical intervention. To determine the appropriate treatment strategy, head computed tomography angiography (CTA) was performed. Given the potential for endovascular therapy, whole-body computed tomography (CT) was additionally performed to assess the feasibility of vascular access. This imaging incidentally revealed a 1.0-cm, well-circumscribed, non-spiculated mass in the right breast. Subsequent mammography, ultrasonography, and core needle biopsy confirmed estrogen receptor-positive invasive ductal carcinoma. The aneurysm was treated first with microsurgical clipping, followed by breast-conserving surgery and adjuvant therapy. The postoperative course was uneventful, and no recurrence of either condition was observed at one-year follow-up. In postmenopausal women with aneurysms that gradually enlarge over many years, careful attention to extracranial regions on available imaging becomes particularly important, as clinically relevant disease can arise silently during long-term surveillance. Systematic evaluation of extracranial regions on preoperative imaging may influence both treatment planning and overall patient management.

## Introduction

Unruptured intracranial aneurysms are often discovered incidentally and may be followed for years before surgical intervention is considered [1]. In older patients, although treatment decisions must consider comorbidities and operative tolerance, enlargement over time is a key determinant in recommending treatment [2,3]. In cross-sectional CT examinations that include adjacent thoracic and abdominal regions, clinically relevant extracranial incidental findings may occasionally be detected [4,5]. In contrast, neurosurgical preoperative evaluation for unruptured cerebral aneurysms is typically focused on intracranial structures, and systemic imaging is not routinely performed. This clinical scenario has been infrequently reported in the literature.

We present a case of an asymptomatic, small, non-spiculated estrogen receptor (ER)-positive breast cancer discovered during preoperative evaluation for a middle cerebral artery (MCA) aneurysm that had demonstrated slow enlargement over 16 years.

## Case presentation

**Figure 1:**
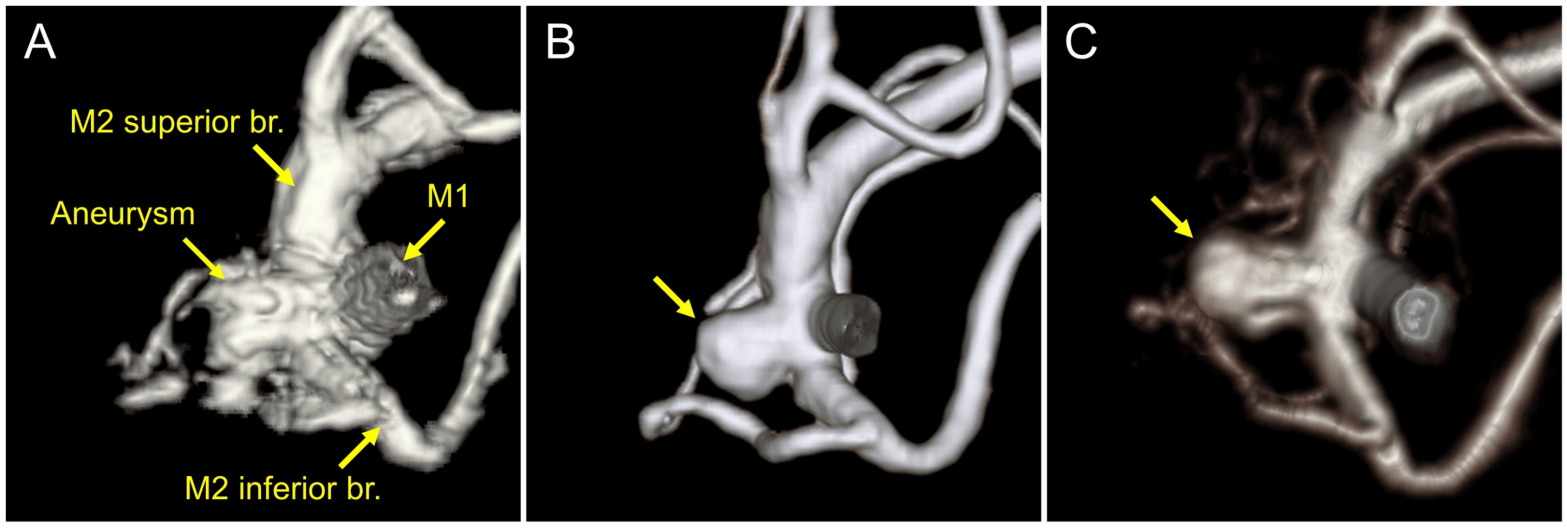
Serial magnetic resonance angiography demonstrating gradual enlargement of the aneurysm over 16 years Initial magnetic resonance angiography showing a small aneurysm at the bifurcation of the right middle cerebral artery (A). Follow-up imaging demonstrating an increase in aneurysm height at six years (yellow arrow, B). Further enlargement of the aneurysm observed at 16 years (yellow arrow, C).

A woman in her 70s was found to have an unruptured cerebral aneurysm at the bifurcation of the right MCA during routine brain screening 16 years ago. She had a history of hypertension treated with antihypertensive medication, in addition to a remote history of treated thyroid cancer. Head magnetic resonance angiography (MRA) was performed every six months during the first three years, and annually thereafter. Initially, the cerebral aneurysm measured 5.1 × 3.7 mm in diameter and 2.8 mm in height (Figure 1A). There was no change in the major or minor diameters, but the height gradually increased, reaching 4 mm at six years (Figure 1B) and exceeding 5 mm at 13 years. Further enlargement was observed at 16 years (Figure 1C). Given the progressive morphological changes, including bleb formation and gradual enlargement, together with patient-related risk factors such as advanced age and hypertension, surgical intervention was deemed appropriate. 

**Figure 2:**
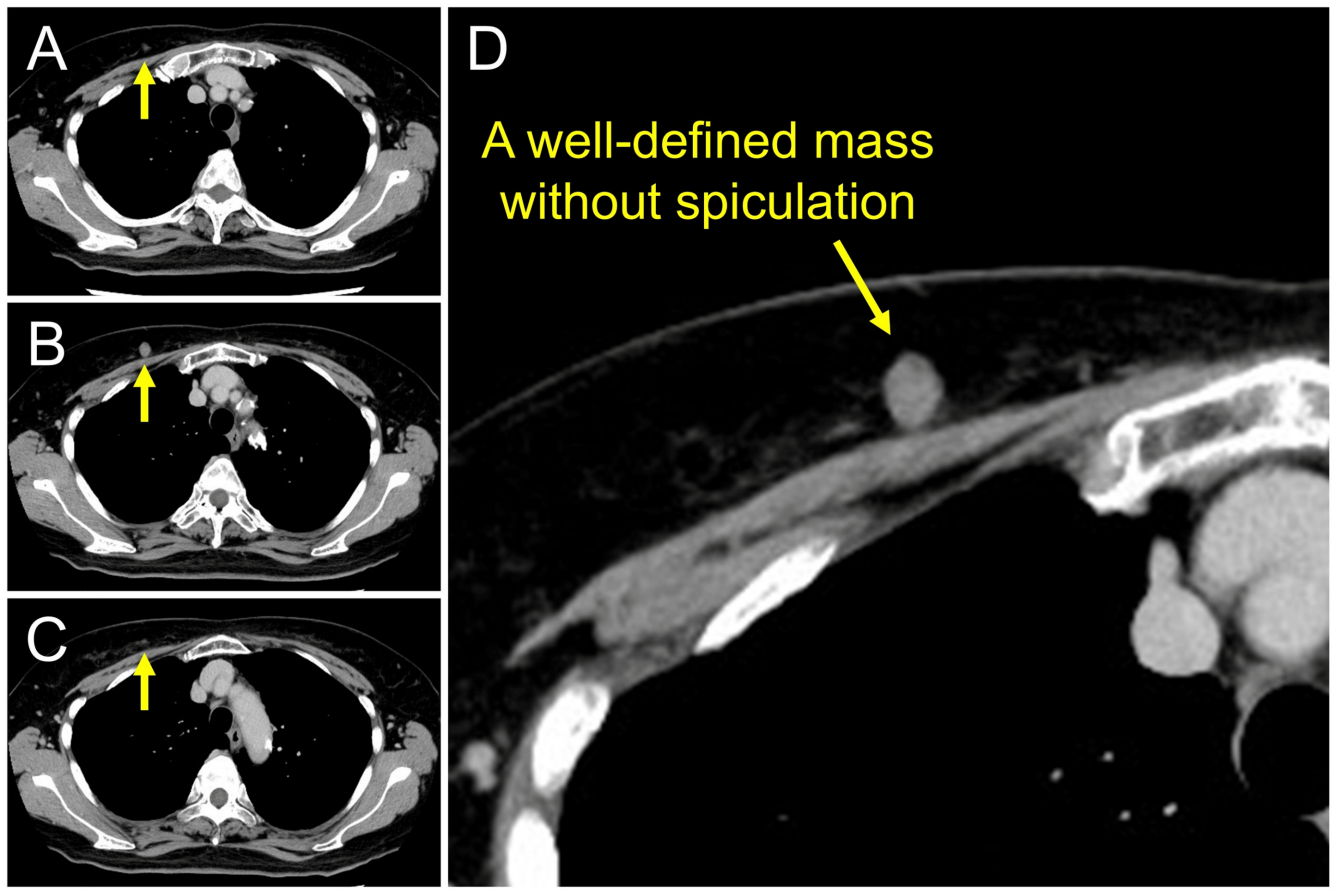
Whole-body computed tomography performed for evaluation of vascular access incidentally revealing a breast lesion Chest CT with 5-mm slice thickness revealed a well-defined mass in the right breast, showing a few malignant features such as spiculation. Serial images across three consecutive slices (A-C) and an enlarged view of the lesion (D) are shown. The yellow arrows in panels A-C indicate the breast mass. The lesion is clearly visible in panel B but only partially visible in panels A and C.

To determine the appropriate treatment strategy, head computed tomography angiography (CTA) and venography were performed to evaluate the cerebral vasculature. Considering the potential for endovascular therapy, whole-body computed tomography (CT) was additionally performed to assess the feasibility of vascular access. This imaging incidentally revealed a 1.0-cm, well-circumscribed, non-spiculated mass in the right breast, located in the A region of the breast (upper outer quadrant) (Figures 2A-2D). The lesion was initially identified on the radiology report and subsequently evaluated with dedicated breast imaging. The patient was referred to breast surgeons, and mammography, ultrasonography, and core needle biopsy confirmed estrogen receptor-positive invasive ductal carcinoma. After discussion with the breast surgeons, it was decided that the cerebral aneurysm would be treated first and the breast cancer subsequently.

**Figure 3:**
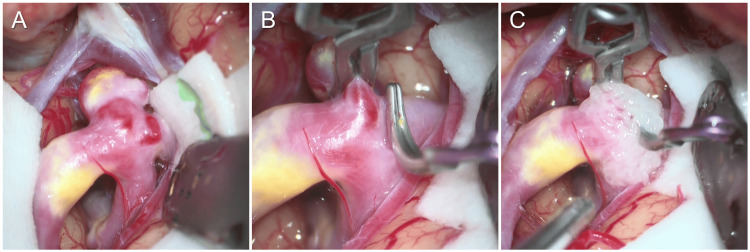
Intraoperative findings during microsurgical treatment of the cerebral aneurysm A saccular aneurysm with a yellowish wall suggestive of arteriosclerotic changes (A). A small bleb was identified near the neck, and both the main aneurysm and the bleb were secured with clips (B). The residual dog-ear portion was reinforced with fibrin glue (C).

Intraoperatively, the cerebral aneurysm was exposed via a standard frontotemporal craniotomy using a transsylvian approach. The aneurysm wall appeared yellow, suggesting arteriosclerotic changes (Figure 3A). The aneurysm was clipped with an FT750T (Aesculap, B. Braun, Tuttlingen, Germany). A small bleb was identified near the neck and was secured with an FT724T clip (Aesculap, B. Braun, Tuttlingen, Germany) (Figure 3B). The dog-ear portion was subsequently reinforced with fibrin glue (Figure 3C). The perioperative course was uneventful, and the patient was discharged home 10 days after surgery.

Following recovery, she began hormone therapy with an aromatase inhibitor, underwent breast-conserving surgery and sentinel lymph node biopsy, and was clinically staged as cT1N0M0. She later received adjuvant radiotherapy. At one-year follow-up after aneurysm surgery, there was no evidence of recurrence of either the cerebral aneurysm or the breast cancer. She remains under regular outpatient follow-up.

## Discussion

To our knowledge, reports specifically addressing incidental extracranial malignancies detected during preoperative evaluation of unruptured intracranial aneurysms remain scarce. In neurosurgical practice, imaging of the trunk is performed only in limited situations. In neuro-oncologic practice, body imaging may be added when newly identified intracranial lesions raise concern for metastasis or extracranial malignancy; however, the clinical utility of such additional imaging remains controversial [6,7]. More recently, in the acute stroke setting, extending head-focused imaging to whole-body CT angiography has been reported to reveal extracranial malignancies and systemic thrombotic disease [8]. By contrast, in the evaluation of unruptured cerebral aneurysms, current guidelines emphasize cerebrovascular imaging - CTA, MRA, and digital subtraction angiography - and do not address the use of routine thoracic or abdominal CT [9,10]. From a clinical standpoint, opportunities to identify extracranial disease may therefore be structurally limited in routine aneurysm-focused evaluation. Patients with long-term follow-up who have no particular comorbidities and appear clinically stable may further reinforce a narrow diagnostic focus on the intracranial lesion. In this case, whole-body CT obtained during vascular access assessment for potential endovascular treatment incidentally revealed an asymptomatic early-stage breast cancer that might otherwise have remained undetected in routine neurosurgical evaluation. As endovascular therapy becomes increasingly prevalent for unruptured aneurysms, the detection of incidental extracranial findings in this setting may be encountered more frequently in clinical practice.

Reported detection rates of incidental breast lesions on CT performed for non-breast indications are typically around 1%-2%, although higher rates of up to 7.6% have been reported, indicating substantial variability across study populations [11-13]. Several retrospective series have shown that a substantial proportion of these lesions are malignant, with reported malignancy rates of approximately 30% [13-15]. Spiculation is among the strongest CT predictors of malignancy, whereas small non-spiculated lesions may appear less suspicious and, therefore, be more easily overlooked on non-breast-focused imaging [14,15]. More recent data further suggest that incidental breast cancers on chest CT are frequently missed, particularly when lesions are sub-centimeter or only weakly enhancing [16]. In the present case, the breast tumor was small (10 mm) and non-spiculated, consistent with the characteristics of lesions that may be underestimated or overlooked unless careful attention is paid to the breast on systemic CT. This case highlights the importance of recognizing that, even when whole-body CT is performed during neurosurgical preoperative evaluation, certain extracranial malignancies may remain difficult to detect unless careful scrutiny is applied.

At first glance, the coexistence of an enlarging unruptured intracranial aneurysm and an early-stage breast cancer may appear coincidental. After menopause, ovarian estrogen production declines substantially, a transition that has been implicated in intracranial aneurysm formation and progression through effects on vascular integrity [17,18], while peripheral conversion of androgens to estrone persists, with an increased likelihood of developing estrogen receptor-positive breast cancer [19,20]. Although no direct mechanistic link between the two diseases has been established, their parallel dependence on age-related hormonal shifts may offer a hypothetical explanation for why they occasionally coexist in older women without other comorbidities.

As this report is based on a single clinical observation, the findings should be interpreted with appropriate caution. At present, no clear criteria exist to guide the routine use of broader systemic imaging in this clinical context. Therefore, it is not intended to advocate indiscriminate CT examinations, particularly in light of concerns regarding radiation exposure. This report underscores the importance of careful clinical judgment when considering treatment in older patients, and the optimal role of systemic imaging remains an important issue for future investigation.

## Conclusions

Postmenopausal women with aneurysms that gradually enlarge over many years may represent a clinical context in which careful attention to extracranial regions on available imaging becomes particularly important, as extracranial disease can arise silently during long-term surveillance. Neurosurgeons should carefully evaluate extracranial regions on preoperative imaging, as such findings may influence treatment planning and overall patient management.
